# Detection of cefotaxime- and enrofloxacin-resistant and *mcr-1*-positive *Escherichia coli* at farm level in younger and older calf groups across different production systems in Germany

**DOI:** 10.1186/s12866-026-05693-2

**Published:** 2026-09-22

**Authors:** Caroline Robé, Jorinde Baer, Roswitha Merle, Lisa Gorisek, Uwe Roesler, Anika Friese

**Affiliations:** 1 Veterinary Centre for Resistance Research, Institute of Animal Hygiene and Environmental Health, Freie Universität Berlin, Robert-von-Ostertag-Straße 8, Berlin, 14163 Germany; 2 Veterinary Centre for Resistance Research, Institute of Veterinary Epidemiology and Biostatistics, Freie Universität Berlin, Berlin, Germany

**Keywords:** Antibiotic resistance, (veal) calf, ESBL, Cefotaxime, Fluoroquinolone, Enrofloxacin, Colistin, Livestock

## Abstract

**Objective:**

Studies on antibiotic resistance in calves rarely differentiate between production systems or younger and older calf groups within farms and commonly rely on non-selective laboratory methods. We investigated the qualitative farm-level detection and genetic diversity of antimicrobial-resistant *E. coli* from calves in different production systems in Germany using selective culture. Production systems included closed dairy farms (A), farms purchasing veal calves < 10 weeks (B), farms purchasing veal calves aged ≥ 10 weeks (C), suckler cow herds (D), and traders/collection points (E).

**Methods:**

Eighty-one farms were sampled. Two pooled fecal samples representing younger and older calf groups, without recording exact ages, were collected from production systems A–C, and one sample each from D and E. Samples were cultured on MacConkey agar supplemented with cefotaxime (CTX), enrofloxacin (ENR) or colistin (COL) for qualitative detection of *E. coli* subpopulations. Recovered isolates were characterized by PCR for mobile ESBL-/pAmpC-associated genes, plasmid-mediated colistin resistance genes, quinolone resistance-determining region substitutions, and plasmid-mediated quinolone resistance genes. Phylogenetic relatedness of selected isolates was assessed by cgMLST-based hierarchical clustering.

**Results:**

In systems A–C and among traders CTX- and ENR-resistant *E. coli* were widespread at farm level (> 70%), whereas suckler cow herds had substantially lower detection rates (< 25%). In systems A–C, resistant *E. coli* were detected more frequently at farm level in younger than older calf groups. Dominant ESBL genes were *bla*_CTX−M−1, −15_ and _− 14_. Common amino acid substitutions associated with quinolone resistance included S83L and D87N/Y/H in GyrA and S80I in ParC, in addition to the detection of the *qnrS* and *aac(6’)-Ib-cr* genes. *Mcr-1-*positive *E. coli* recovered from COL-supplemented agar were detected less frequently (< 20%) in all production systems examined. Analysis of two samples from production systems A–C identified highly related isolates (HC10) within individual farms but not between farms.

**Conclusion:**

Differences in the qualitative farm-level detection and genetic diversity of CTX- and ENR-resistant and *mcr-1*-positive *E. coli* subpopulations were observed between production systems and between younger and older calf groups within farms. These findings suggest that production system and sampled calf group should be considered in the surveillance of antibiotic-resistant *E. coli* in calves.

**Supplementary Information:**

The online version contains supplementary material available at https://doi.org/10.1186/s12866-026-05693-2.

## Background

Antibiotic-resistant bacteria are frequently detected in calves [1–3]. However, existing studies and surveillance programs in Germany and Europe do not specifically address the different veal calf production systems. Antibiotic resistance in veal calves is monitored at the national and European level due to their importance in livestock production, with commensal *Escherichia coli* (*E. coli*) used to monitor the development of resistance [4, 5]. According to the German surveillance program of 2023, only 50% of the commensal *E. coli* examined from cecal samples of veal calves at slaughterhouses were susceptible to all antibiotic classes tested, and 25% of the isolates tested showed resistance to three or more antibiotic classes [5]. To reduce the risk of antibiotic resistance development and spread in fattening animals, including veal calves, Germany introduced an antimicrobial use minimization system in 2014. No significant reduction in antibiotic use in veal calves was observed after implementation of the German antimicrobial minimization system. One explanation proposed for this finding is the broad classification of ‘veal calves’, as farms differ considerably in terms of calves’ age, origin, husbandry, management, and antibiotic treatment [6]. Studies have shown that the use of antibiotics is particularly high in young veal calves, which also correlates with high resistance rates in *E. coli* in these animals [3, 7, 8]. However, the available data are largely based on non-selective laboratory methods, which may underestimate resistant *E. coli* subpopulations. In particular, resistance to critically important antimicrobials as defined by the World Health Organization [9], such as fluoroquinolones and colistin, is often detected at low levels and may therefore be underestimated in surveillance programs. More differentiated data on resistance to third-generation cephalosporins are available from selective laboratory methods, with two-thirds of cecal samples from veal calves at slaughterhouse level testing positive for extended-spectrum beta-lactamase- (ESBL-) and plasmid-mediated AmpC beta-lactamase- (pAmpC-) producing *E. coli* in Germany [5]. Moreover, scientific studies have shown high detection rates of ESBL-producing *E. coli* in veal calves, but these studies have focused on individual farms or production systems, which do not allow for comprehensive conclusions to be drawn [10–13]. The aim of this descriptive study was to investigate the qualitative farm-level detection and genetic diversity of CTX- and ENR-resistant *E. coli* and *mcr*-positive *E. coli* recovered on COL-supplemented agar in pooled fecal samples from veal calves representing different production systems and younger and older calf groups within farms using selective culture methods. Dairy farms were also included because calves are raised not only for herd replacement but also for meat production. Investigating the qualitative farm-level detection and genetic diversity of resistant *E. coli* may provide a basis for improved surveillance of antimicrobial resistance in *E. coli* and, consequently, for targeted measures to reduce antibiotic resistance in calf production.

## Materials and methods

### Study farms

Eighty-one calf farms from four different production systems (A-D) and traders (E) were visited between 2019 and 2021. The production systems were classified according to their calf sourcing strategy and, where applicable, the age at which calves were purchased. Production system A comprised 17 dairy farms with a closed farming system without regular purchase of calves. Production system B comprised 33 farms that purchase calves under 10 weeks of age. The 15 farms that purchased calves aged 10 weeks or older were classified as production system C. Production system D comprised calves from nine suckler cow herds with closed herd management, where calves were kept with their mother cows. With the exception of production system A, where it was not possible to distinguish between calves intended for herd replacement (milk production) and those for fattening (meat production), all animals sampled were fattening calves. In addition, seven traders and collection points (E), where calves from different farms of origin were temporarily collected and regrouped before transport to fattening farms, were examined. The investigated farms were located in northern Germany (Lower Saxony, North Rhine-Westphalia, and Schleswig-Holstein), eastern Germany (Brandenburg, Mecklenburg-Western Pomerania, and Thuringia), and southern Germany (Bavaria) as described in Merle et al. [7].

### Sampling on the farms

On farms of production system A-C, two pooled fecal samples, each consisting of ten fresh fecal droppings, were collected from a younger and an older calf group. Only one pooled fecal sample was taken from calves from suckler cow herds (D) and from traders/collection points (E). The exact age of the calves was not recorded because the sampling design was based on a relative within-farm classification into younger and older calf groups, which was determined together with the farmer on each farm. This classification was used to compare the two calf groups within each farm and to determine which group should be sampled for the qualitative farm-level detection of resistant *E. coli*. Recording exact ages was not considered appropriate for this purpose because the age structure of calf groups varied considerably between farms. The pooled fecal samples were collected from the floor without any direct contact with the animals kept in routine livestock production. The samples were collected with the farm owner’s consent, and no approval from an institutional or national ethics committee was required for this study.

### Laboratory methods

#### Sample preparation and species identification

Twenty grams of pooled feces were mixed with 180 mL Luria Bertani broth (LB; Carl Roth, Karlsruhe, Germany) and homogenized in a stomacher for two minutes (200 rounds per minute). Following, 1:10 dilution series were performed and 100 µL of appropriate dilutions were plated on MacConkey Agar No. 3 (Oxoid, Wesel, Germany). For the qualitative detection of resistant *E. coli* subpopulations, each sample was cultured on three MacConkey agar plates supplemented with 1 µg/mL cefotaxime (CTX, AppliChem, Darmstadt, Germany), 2 µg/mL enrofloxacin (ENR, Sigma- Aldrich, Steinheim, Germany) or 2 µg/mL colistin sulfate (COL, Carl Roth, Karlsruhe, Germany), and incubated for 18–24 h at 37 °C. The antibiotic concentrations were selected based on their demonstrated suitability for the selective recovery of *E. coli* from fecal samples [14]. In cases with no growth, homogenized fecal samples were incubated for 18–24 h at 37 °C, and 10 µL were streaked out onto the antibiotic-supplemented agar plates described above to lower the detection limit for qualitative detection. A maximum of three *E. coli* isolates with distinct colony morphologies per supplemented agar plate and sample were subcultured on a second agar plate with identical antibiotic supplementation and incubated for 18–24 h at 37 °C (maximum of nine *E. coli* morphologies per sample). Colonies were confirmed by matrix-assisted laser desorption time of flight (MALDI-TOF, MALDI Microflex LT^®^ and Biotyper database^®^; Bruker Daltonics, Bremen, Germany) and stored at -80 °C in LB containing 20% glycerol (Carl Roth, Karlsruhe, Germany) for further analyses.

#### Molecular biological analyses

DNA was extracted from all *E. coli* isolates recovered from the respective CTX-, ENR-, or COL-supplemented MacConkey agar plates using the boiling method [15] and subjected to PCR. Only isolates in which the corresponding resistance determinants were detected were included in the subsequent analyses. CTX-resistant isolates were investigated in a real-time PCR for *bla*_CTX−M_, *bla*_SHV_, *bla*_TEM_, and *bla*_CMY_ as published by Roschanski et al. [15] and sequenced according to Projahn et al. [16]. COL-selected isolates were analyzed for the transferable colistin resistance genes *mcr-1* to *mcr-5* according to Rebelo et al. [17]. ENR-resistant isolates were analyzed for mutations in the quinolone resistance-determining regions (QRDRs) of DNA gyrase (*gyrA*) and topoisomerase IV (*parC*) genes, and the corresponding amino acid substitutions in GyrA and ParC were determined as published by Bansal et al. [18]. Additionally, isolates were screened for plasmid-mediated quinolone resistance (PMQR) genes encoding for (i) target protection mediated by qnr genes (*qnrA*, *qnrB*,* qnrC*,* qnrD* and *qnrS*), (ii) antibiotic modification mediated by *aac(6´)-Ib-cr*, and (iii) quinolone efflux pumps (*qepA*,* oqxA* and *oqxB*) with previously described primers [19–26]. Phylogenetic typing was performed according to Clermont et al. [27] with modifications published in Projahn et al. [16] for all confirmed antibiotic-resistant *E. coli* isolates.

#### Whole-genome sequencing

To investigate the phylogenetic relatedness of resistant *E. coli* isolates, a selected subset of 62 CTX- and ENR-resistant isolates from production systems A-C was subjected to whole-genome sequencing. Isolates were selected from farms in which both investigated samples yielded CTX- and/or ENR-resistant *E. coli* with identical resistance determinants and phylogenetic groups (13 farms for CTX-resistant *E. coli* and 18 farms for ENR-resistant *E. coli*). The Invitrogen PureLink Genomic DNA Mini Kit (Thermo Fisher Scientific, Wesel, Germany) was used for DNA isolation and isolates were whole-genome sequenced on an Illumina NextSeq (300-bp paired-end, coverage between 80x and 100x; LGC Genomics GmbH, Berlin, Germany). The data pre-processing and de novo assembly of raw reads was performed with EnteroBase [28]. Under the BioProject Accession number PRJNA1454735 (http://www.ncbi.nlm.nih.gov/bioproject/1454735) in the NCBI Sequence Read Archive the *E. coli* sequences are publicly available. Genotypic antimicrobial resistance characterization, phylogenetic analysis, and Hierarchical Clustering (HierCC) of *E. coli* strains based on core-genome multi-locus sequence typing (cgMLST) were performed using EnteroBase [29–31]. Isolates belonging to the same HC10 cluster, defined by a maximum pairwise distance of 10 alleles, were considered highly related [32].

### Statistical analysis

Farm-level detection rates were calculated as percentages with corresponding 95% confidence intervals (95% CIs) for binomial proportions using the Wilson score method.

## Results

We investigated CTX- and ENR-resistant *E. coli* and *mcr*-positive *E. coli* recovered on COL-supplemented agar in pooled fecal samples from calves kept on farms with different production systems. Two pooled fecal samples representing younger and older calf groups within each farm were collected (A-, B-, and C-farms; one sample from D-, and E-farms) to compare the farm-level detection rates, resistance genes, and phylogenetic relationship of isolates.

### Farm-level detection rates and antimicrobial resistance genes

**Fig. 1:**
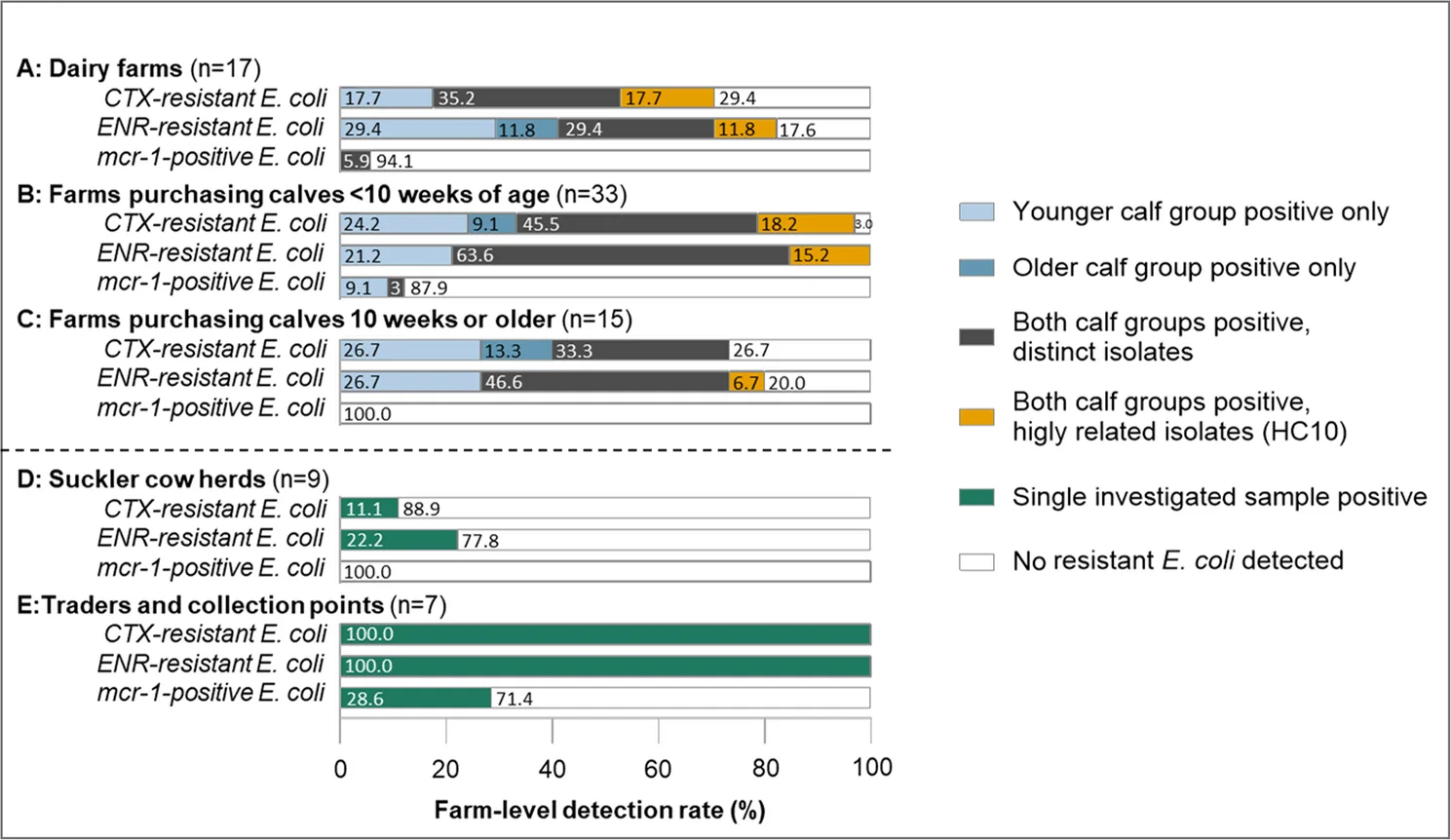
Farm-level detection rates (%) of cefotaxime (CTX)- and enrofloxacin (ENR)-resistant *E. coli* and *mcr-1*-positive *E. coli* recovered from colistin supplemented agar in pooled fecal samples from different calf production systems in Germany. Two pooled fecal samples representing younger and older calf groups were collected from each farm in production systems **A**-**C**, whereas only one pooled fecal sample was collected from production systems **D** and **E**. The number of investigated farms was 17 (**A**), 33 (**B**), 15 (**C**), 9 (**D**), and 7 traders/collection points (**E**). Phylogenetic relationships were assessed by cgMLST-based hierarchical clustering. Both samples positive, distinct isolates: both samples from the same farm yielded resistant *E. coli* with different resistance determinants and/or phylogenetic groups, or isolates with identical resistance determinants and phylogenetic groups differed by more than 10 alleles; Both samples positive, highly related isolates: both samples yielded resistant *E. coli* isolates differing by a maximum of 10 alleles (HC10) and were therefore considered highly related. The dashed line separates production systems **A**-**C** (two pooled fecal samples per farm) from production systems **D**-**E** (one pooled fecal sample per farm/location)

High farm-level detection rates of CTX- and ENR-resistant *E. coli* were observed in production systems A, B, and C. CTX- and ENR-resistant *E. coli* were detected in 97.0% (32/33, 95%-CI 84.7–99.5%) and 100% (33/33, 95%-CI 89.6–100.0%) of the group B farms investigated. Compared to group B, lower farm-level detection rates were observed in group A with 70.6% CTX (12/17, 95%-CI 46.9–86.7%) and 82.4% ENR (14/17, 95%-CI 59.0–93.8%), and in group C, with 73.3% CTX (11/15, 95%-CI 48.1–89.1%) and 80.0% ENR (12/15, 95%-CI 54.8–93.0%), respectively. The farm-level detection rate was lowest among the farms in group D, at 11.1% CTX (1/9, 95%-CI 2.0–43.5%) and 22.2% ENR (2/9, 95%-CI 6.3–54.7%, only one pooled fecal sample was investigated per farm). All seven investigated traders/collection points tested positive for CTX- and ENR-resistant *E. coli* (7/7, 95%-CI 64.6–100.0%, only one pooled fecal sample per trader/collection point was investigated (Fig. 1, Supplementary Tables 1 and 2)). The resistance genes detected in the phenotypically CTX-resistant isolates were *bla*_CTX−M−1, −14, −15, −27, −55, −65, −211_ and *bla*_SHV−12_, with *bla*_CTX−M−1, −14, −15_ being the most frequently detected (Supplementary Tables 1 and 3). Amino acid substitutions in the QRDRs of GyrA (S83L and D87N/Y/H) and ParC (A56T, S57T, S80I, and E84A/G/K) were detected in phenotypically ENR-resistant isolates (Supplementary Tables 2 and 3). Five ENR-resistant isolates harbored only the GyrA substitution S83L without an amino acid substitution in ParC (two from group A farms and three from group B farms). The detected PMQR genes were *qnrS* and *aac(6´)-Ib-cr*, with *qnrS* identified in all isolates harboring only the GyrA amino acid substitution S83L. All *E. coli* isolates recovered from COL-supplemented agar carried the *mcr-1* gene. The farm-level detection rate of *mcr-1*-positive *E. coli* was lower than that of CTX- and ENR-resistant *E. coli* in all production systems and among all traders. The farm-level detection rate of *mcr-1*-positive *E. coli* was 5.9% (1/17, 95%-CI 1.1–27.0%) in group A farms, 12.1% (4/33, 95%-CI 4.8–27.3%) in group B farms, 28.6% (2/7, 95%-CI 8.2–64.1%) among traders and collection points, and 0% in group C (0/15, 95%-CI 0.0–20.4%) and D farms (0/9, 95%-CI 0.0–29.9%). At the isolate level, B1 (*n* = 114) and A/C (*n* = 103) were the predominant phylogenetic groups, followed by A (*n* = 30), E (*n* = 14), F (*n* = 12), E/D (*n* = 4), and C (*n* = 1), while B2 was not detected (Supplementary Table 3).

### Detection of antimicrobial-resistant *E. coli* in younger and older calf groups

In production systems A, B, and C, higher farm-level detection rates of CTX- and ENR-resistant *E. coli* were detected in the pooled fecal samples from younger than older calf groups within the same farm (Fig. 1, Supplementary Tables 1 and 2). CTX-resistant *E. coli* were detected exclusively in the older calf group in 9.1% (3/33, 95%-CI 3.1–23.6%) of group B farms and 13.3% (2/15, 95%-CI 3.7–37.9%) of group C farms. Similarly, ENR-resistant *E. coli* were detected exclusively in the older calf group in 11.8% (2/17, 95%-CI 3.3–34.3%) of group A farms. In all remaining farms, as well as for *mcr-1*-positive *E. coli*, resistant isolates were detected either in both samples or exclusively in the younger calf group, resulting in higher farm-level detection rates for CTX- and ENR-resistant and *mcr-1*-positive *E. coli* when younger calves were sampled. When both investigated samples from the same farm were positive for CTX- or ENR-resistant *E. coli*, distinct isolates carrying different resistance determinants and/or belonging to different phylogenetic groups were detected in most cases in the younger and older calf groups of production systems A-C (Supplementary Table 3).

### Genetic relationship of antimicrobial-resistant *E. coli* on farms

Among production systems A-C, identical resistance determinants and phylogenetic groups were identified in both positive fecal samples from 13 farms for CTX-resistant *E. coli* (group A: 29.4% (5/17, 95%-CI 13.3–53.1%), group B: 24.2% (8/33, 95%-CI 12.8–41.0%), group C: 0.0% (0/15, 95%-CI 0.0–20.4%)) and from 18 farms for ENR-resistant *E. coli* (group A: 29.4% (5/17, 95%-CI 13.3–53.1%), group B: 27.3% (9/33, 95%-CI 15.1–44.2%), group C: 26.7% (4/15, 95%-CI 10.9–52.0%)). Whole-genome sequencing identified highly related isolates (HC10; ≤ 10 allele differences, Supplementary Table 4) in 9 of the 13 selected farms with CTX-resistant *E. coli* (group A: 17.7% (3/17, 95%-CI 6.2–41.0%), group B: 18.2% (6/33, 95%-CI 8.6–34.4%)). In the remaining four farms, isolates differed by more than 10 alleles and were therefore classified as distinct isolates (Fig. 1). For ENR-resistant *E. coli*, highly related isolates were identified in 8 of the 18 selected farms (group A: 11.8% (2/17, 95%-CI 3.3–34.3%), group B: 15.2% (5/33, 95%-CI 6.7–30.9%), group C: 6.7% (1/15, 95%-CI 1.2–29.8%)); isolates from the remaining ten farms were classified as distinct isolates. For *mcr-1*-positive *E. coli*, no isolates with identical resistance genes and phylogenetic groups were detected in any of the farms investigated. No phylogenetically related isolates were detected between farms within the selected subset of whole-genome sequenced isolates. 

## Discussion

This study investigated the qualitative farm-level detection and genetic diversity of CTX- and ENR-resistant and *mcr-*positive *E. coli* in pooled fecal samples from calves representing different production systems in Germany. Overall, high farm-level detection rates of CTX- and ENR-resistant *E. coli* were observed in production systems A-C and among traders/collection points (E), whereas substantially lower detection rates were found in suckler cow herds (D). The comparatively low detection rates observed in suckler cow herds may be associated with differences in herd management compared with the other production systems, but may also have been influenced by the collection of only one pooled fecal sample per farm and the relatively small number of investigated herds. In comparison, only one pooled fecal sample was also collected from each of the seven investigated traders/collection points, yet CTX- and ENR-resistant *E. coli* were detected at all locations. Suckler calves are typically reared together with their mother cows in closed herds without regular calf purchase, which may reduce the introduction and circulation of resistant *E. coli*. Differences in antimicrobial use between production systems may also contribute to the observed findings. However, antimicrobial use and specific management factors were not investigated in the present study and should therefore be considered possible explanations.

Previous studies comparing antimicrobial resistance in conventional and organic production have reported inconsistent results. In Switzerland, no ESBL-producing *E. coli* were detected in organically reared dairy calves, whereas ESBL-producing *E. coli* were detected in conventionally reared dairy calves and resistance to some individual antibiotics was reported to be higher in organic than in conventional farms [33]. In contrast, a Swedish study found no differences in antibiotic resistance in *E. coli* between organic and conventional dairy calves and attributed this to the generally low antibiotic use in both production systems [34]. Although the suckler cow herds investigated in our study cannot be equated with organic production systems, these studies illustrate that differences in husbandry and management may be relevant when interpreting antimicrobial resistance patterns in *E. coli* across calf production systems. Comparisons with organic production systems should also consider that there is no uniform definition of organic farming [35].

Traders and collection points collect calves from different farms of origin and regroup them according to age before transportation to fattening farms. Current veal calf production practices, including regrouping followed by national and international calf trade, have been discussed as contributing to the high occurrence of resistant bacteria in young veal calves. Regrouping and transportation are associated with increased stress, and young veal calves frequently receive metaphylactic (group-wise) antibiotic treatment to prevent respiratory and gastrointestinal diseases during the fattening period [3, 10, 36, 37]. Antimicrobial treatment exerts selective pressure on bacterial populations and thereby promotes the selection of resistant bacteria [38]. These factors may therefore contribute to the high detection rates observed at traders/collection points and in the production systems purchasing young veal calves in our study. The relationship between antibiotic use and resistance is further supported by national surveillance data from European countries, showing declining resistance rates in different animal species alongside reductions in antibiotic consumption over the last decade, although these trends have stabilized in recent years [39, 40]. Our findings support the relevance of a more differentiated consideration of veal calf production systems. The broad classification of ‘veal calves’ has previously been discussed as a possible explanation for the lack of a significant reduction in antibiotic use following the introduction of the German antibiotic minimization concept, as farms differ considerably in calf age, origin, keeping, management, and antibiotic treatment [6]. To date, only a few studies have investigated the relationship between antibiotic use and antibiotic resistance in veal calves, and these studies are limited in scope and have inconsistent findings [1, 7, 10, 41].

Previous studies have shown higher resistance rates in young compared with older veal calves [1, 11, 12, 42]. Our findings are consistent with these observations, as CTX- and ENR-resistant *E. coli* were detected more frequently at farm level in pooled fecal samples from the younger than from the older calf groups in production systems A-C. Farm-level detection rates were highest in production system B, comprising farms that purchase veal calves under 10 weeks of age. Thus, our results indicate that sampling the younger calf group increases the likelihood of detecting antimicrobial-resistant *E. coli* at the farm level. However, as the exact age of the calves was not recorded, our findings reflect differences between younger and older calf groups within individual farms rather than generalizable age-specific effects. As the actual age of calves may differ considerably between production systems and farms, studies recording the exact age of the sampled calf groups are needed to characterize age-dependent differences more precisely. At the time of our study, calves could be transported from 14 days of age and were commonly moved from their farm of birth via collection points to fattening farms. As young animals are particularly susceptible to disease due to their immature immune system, the minimum transport age in Germany was increased from 14 to 28 days in 2022 [43–45]. This change might positively affect calf health [46] and potentially contribute to reducing the development of antimicrobial resistance, although this was not investigated in our study.

When investigating two pooled fecal samples representing different calf age groups from farms in production systems A-C, we detected considerable diversity in resistance determinants among CTX- and ENR-resistant *E. coli* subpopulations. Our findings indicate that examining only one calf group may not adequately reflect the diversity of resistance determinants present on a farm. However, as only two pooled fecal samples were collected per farm and a maximum of three distinct *E. coli* colony morphologies were selected per selective agar plate, our study does not allow recommendations on the number of samples or isolates required to characterize resistance diversity on calf farms. Bosman et al. [47] recommended a sampling strategy comprising 20 individual pooled fecal samples from veal calves and the investigation of 90 isolates for phenotypic resistance. While this approach provides more extensive sampling, it involves high workload and does not consider calf age or housing system. Identical resistance determinants and highly related isolates (HC10) in different calf groups were predominantly detected on dairy farms with a closed housing system and on farms purchasing calves under 10 weeks of age. An HC10 cluster, representing *E. coli* isolates with a maximum pairwise distance of 10 cgMLST alleles, is commonly interpreted as being consistent with short-term transmission from a common source [32]. However, only a selected subset of resistant *E. coli* isolates was subjected to whole-genome sequencing, comprising isolates with identical resistance determinants and phylogenetic groups in both samples from the same farm. The cgMLST findings therefore refer to this selected subset and cannot be generalized to the entire isolate collection. In closed dairy farms, the occurrence of identical resistance determinants or highly related isolates might be related to the absence of regular calf trade and the within-farm circulation of specific resistance plasmids or resistant strains. Feeding waste milk may provide another explanation, as calves can receive waste milk originating from the same diseased cows, potentially exposing different calf groups to the same resistant bacteria or resistance determinants [48–50]. On farms purchasing calves under 10 weeks of age, the frequent detection of resistant *E. coli* may increase the probability of detecting identical resistance determinants or highly related isolates in different calf groups compared with farms purchasing older calves. Calves sold at an older age may receive more intensive early-life care on their farm of origin, resulting in fewer antibiotic treatments and lower carriage of resistant bacteria. Farmers may invest less in calves intended for sale, whereas optimized early-life care can reduce the risk of infection [8].

No highly related *mcr-1*-positive *E. coli* isolates were identified on the investigated farms, which may reflect their comparatively low farm-level detection rates and is consistent with the generally low detection of *mcr-1* reported in current studies [1, 51]. Compared with studies by Gay et al. [1] and Um et al. [51], which also reported *mcr-3*, only *mcr-1* was detected in our study. The resistance genes identified in CTX-resistant *E. coli* isolates were consistent with those reported in recent European studies on veal calves and further support the frequent detection of CTX-resistant *E. coli* in veal calf production [1, 11, 51, 52]. In contrast, studies characterizing ENR-resistant *E. coli* in livestock are scarce, although fluoroquinolone resistance is relevant for describing the current resistance situation in livestock production and previous studies have also reported co-resistance to fluoroquinolones and third-generation cephalosporins [13, 53]. The resistant *E. coli* isolates from our study showed a diverse phylogenetic background, with B1 and A/C as the predominant phylogenetic groups and no detection of B2, in line with similar distributions reported in previous studies on veal calves [1, 11].

## Conclusion

High farm-level detection rates of CTX- and ENR-resistant *E. coli* were observed in pooled fecal samples from calves across different production systems in Germany, whereas *mcr-1* positive *E. coli* were detected less frequently. Detection rates differed between production systems, with substantially lower rates observed in suckler cow herds. In production systems A-C, sampling younger calf groups generally increased the likelihood of detecting antibiotic-resistant *E. coli* at the farm level, although exact calf ages were not recorded. Our findings highlight the importance of considering production systems and the sampled calf group in the surveillance of antimicrobial-resistant *E. coli*. A more differentiated classification of veal calves, including their production background and age at transport to fattening farms, may improve surveillance of antimicrobial resistance in *E. coli* and provide a basis for targeted measures to reduce antibiotic use and antibiotic resistance in calf production.

## Supplementary Information

Supplementary Material 1: Table S1. Farm-level detection (%) of CTX-resistant *E. coli* and detected combinations of extended-spectrum beta-lactamases in pooled fecal samples from younger and older calf groups across different production systems.

Supplementary Material 2: Table S2. Farm-level detection of ENR-resistant *E. coli* (detected amino acid substitutions in the quinolone resistance determining regions (QRDRs) of DNA gyrase (GyrA) and topoisomerase IV (ParC) together with plasmid-mediated quinolone resistance (PMQR) gene combinations) and *mcr-1*-positive *E. coli* in pooled fecal samples from younger and older calf groups across production systems.

Supplementary Material 3: Table S3. Detailed information on detected cephalosporin-, enrofloxacin- and mcr- resistance genes and phylogenetic group per *E. coli* isolate from calves of different production systems in Germany.

Supplementary Material 4: Table S4. Characterization of whole genome sequenced *E. coli* isolates from calves of different production systems in Germany.

## Data Availability

The datasets supporting the conclusions of this article are included within the article (and its additional files). Under the BioProject Accession number PRJNA1454735 (http://www.ncbi.nlm.nih.gov/bioproject/1454735) in the NCBI Sequence Read Archive the *E. coli* sequences are publicly available.
