# Supplementary material for: Detection of cefotaxime- and enrofloxacin-resistant and mcr-1-positive Escherichia coli at farm level in younger and older calf groups across different production systems in Germany
Source: BMC Microbiol. 2026 Sep 22;26:827. doi: 10.1186/s12866-026-05693-2 (PMC13595704; doi:10.1186/s12866-026-05693-2)
Supplement: Supplementary file 1 [file 12866_2026_5693_MOESM1_ESM.docx]

Supplementary Table 1: Farm-level detection (%) of CTX-resistant *E. coli* and detected combinations of extended-spectrum beta-lactamases in pooled fecal samples from younger and older calf groups across different production systems.

| Sample | Pos. | CTX-M-1 | CTX-M-14 | CTX-M-15 | CTX-M-27 | CTX-M-55 | CTX-M-65 | CTX-M-211 | SHV-12 | CTX-M-1/14 | CTX-M-1/15 | CTX-M-1/211 | CTX-M-14/15 | CTX-M-14/55 | CTX-M-15/27 | CTX-M-15/65 | CTX-M-15, SHV-12 | CTX-M-1/14/15 | CTX-M-1/15/27 |
| --- | --- | --- | --- | --- | --- | --- | --- | --- | --- | --- | --- | --- | --- | --- | --- | --- | --- | --- | --- |
| Prod. sys. A | 70.6% | 41.2% | 0.0% | 17.7% | 0.0% | 0.0% | 5.9% | 0.0% | 0.0% | 0.0% | 17.7% | 0.0% | 0.0% | 0.0% | 0.0% | 0.0% | 0.0% | 0.0% | 0.0% |
| Younger gr. | 70.6% | 29.4% | 0.0% | 17.7% | 0.0% | 0.0% | 5.9% | 0.0% | 0.0% | 0.0% | 17.7% | 0.0% | 0.0% | 0.0% | 0.0% | 0.0% | 0.0% | 0.0% | 0.0% |
| Older gr. | 53.0% | 35.2% | 0.0% | 5.9% | 0.0% | 0.0% | 5.9% | 0.0% | 0.0% | 0.0% | 5.9% | 0.0% | 0.0% | 0.0% | 0.0% | 0.0% | 0.0% | 0.0% | 0.0% |
| Prod. sys. B | 97.0% | 45.5% | 6.0% | 30.3% | 3.0% | 0.0% | 0.0% | 3.0% | 6.1% | 3.0% | 18.2% | 3.0% | 3.0% | 3.0% | 0.0% | 0.0% | 6.1% | 0.0% | 3.0% |
| Younger gr. | 87.8% | 36.4% | 3.0% | 12.1% | 3.0% | 0.0% | 0.0% | 3.0% | 3.0% | 0.0% | 15.2% | 3.0% | 3.0% | 3.0% | 0.0% | 0.0% | 0.0% | 0.0% | 3.0% |
| Older gr. | 72.6% | 24.2% | 3.0% | 24.2% | 0.0% | 0.0% | 0.0% | 0.0% | 6.1% | 3.0% | 3.0% | 3.0% | 0.0% | 0.0% | 0.0% | 0.0% | 6.1% | 0.0% | 0.0% |
| Prod. sys. C | 73.3% | 33.3% | 13.3% | 20.0% | 0.0% | 6.7% | 0.0% | 0.0% | 6.7% | 6.7% | 6.7% | 0.0% | 0.0% | 0.0% | 0.0% | 6.7% | 0.0% | 0.0% | 0.0% |
| Younger gr. | 60.2% | 20% | 0.0% | 6.7% | 0.0% | 6.7% | 0.0% | 0.0% | 6.7% | 6.7% | 6.7% | 0.0% | 0.0% | 0.0% | 0.0% | 6.7% | 0.0% | 0.0% | 0.0% |
| Older gr. | 46.6% | 13.3% | 13.3% | 13.3% | 0.0% | 0.0% | 0.0% | 0.0% | 0.0% | 0.0% | 6.7% | 0.0% | 0.0% | 0.0% | 0.0% | 0.0% | 0.0% | 0.0% | 0.0% |
| Prod. sys. D | 11.1% | 0.0% | 11.1% | 0.0% | 0.0% | 0.0% | 0.0% | 0.0% | 0.0% | 0.0% | 0.0% | 0.0% | 0.0% | 0.0% | 0.0% | 0.0% | 0.0% | 0.0% | 0.0% |
| Prod. sys. E | 100% | 71.4% | 0.0% | 14.3% | 0.0% | 0.0% | 0.0% | 0.0% | 0.0% | 14.3% | 0.0% | 0.0% | 0.0% | 0.0% | 0.0% | 0.0% | 0.0% | 0.0% | 0.0% |

Production systems (Prod. sys.) comprised A (17 dairy farms with a closed farming system without regular purchase of calves), B (33 farms purchasing calves under 10 weeks of age), C (15 farms purchasing calves aged 10 weeks or older), D (9 suckler cow herds with closed herd management), and E (7 traders/collection points). Two pooled fecal samples representing younger and older calf groups were collected from each farm in production systems A–C, whereas only one pooled fecal sample was collected from each farm/location in production systems D and E. A maximum of three *E. coli* isolates with distinct colony morphologies were selected per selective agar plate. Farm-level detection combines the results obtained from the younger and older calf groups within each farm in production systems A–C.
